# The Human Race

**Published:** 1886-04

**Authors:** 


### THE HUMAN RACE.

Some one recently made publicly the remark that if the whole human race were divided into families of five persons each, the State of Texas is large enough to supply half an acre of land to each family. The statement surprised some people, and not a few of them declared hastily, without performing the necessary calculations, that it could not be true.

But it is true. There is supposed to be about fourteen hundred million persons living on the globe. A half acre to each family of five would require one acre for ten persons, or one hundred and forty million acres in all. The area of Texas is more than two hundred and sixty-two thousand square miles, or nearly one hundred and eighty-eight million acres.

Consequently there would be a surplus of almost twenty-eight millions left over after every family was provided for, which would be sufficient for four or five times the present population of the United States.

Not many people realize how little space would be needed to accommodate the whole human race, assembled in one place. Suppose we were to fancy every human being forming one of a vast congregation, seated in fourteen hundred million easy chairs, each occupying a square yard of ground space.

As there are nearly three million one hundred thousand square yards in a square mile, that number represents the size of the congregation that could be seated upon it under the conditions named; and the whole human family could be gathered on a tract of four hundred and fifty-two square miles,—or twenty-one and a quarter miles each way.

Less than two-fifths of the area of the little State of Rhode Island would suffice to give comfortable seating room to the whole human race. One-twelfth of the area of Rhode Island would be enough to afford standing room,—as people stand in a crowd, without crushing, to every man, woman and child on the face of the globe.

**A New Remedy for Hiccough.**—Dr. O. T. Shultz, of Mt. Vernon, Indiana, (*American Practitioner*,) relates a case of hiccough of nine days' duration, which yielded on the morning of the tenth day to one drop of a one per cent. solution of nitro-glycerine, and repeated in one hour. The remedy was continued at intervals of two hours—gradually extended. On the twelfth day permanent relief was obtained, and treatment discontinued.

